# Supplementary material for: Shale oil production and groundwater: What can we learn from produced water data?
Source: PLoS One. 2021 Apr 30;16(4):e0250791. doi: 10.1371/journal.pone.0250791 (PMC8087075; doi:10.1371/journal.pone.0250791)
Supplement: S1 Fig — Note: The author produced the figure in R. The t-tests for the difference in means suggest that the increase in the level of concentration is statistically significant for all four constituents. The null hypothesis of no difference is rejected at the 95% confidence level in all cases (p values are 0.0012, 0.0001, 0.0513, 0.0062, respectively). (DOCX) [file pone.0250791.s001.docx]

**Supplementary Information**

**Shale oil production and groundwater: What can we learn from produced water data?**

**Haoying Wang***

Department of Business and Technology Management, New Mexico Tech, Socorro, NM, USA

Email: [haoying.wang@nmt.edu](mailto:haoying.wang@nmt.edu) (HW)

Supplementary Figures and Tables:


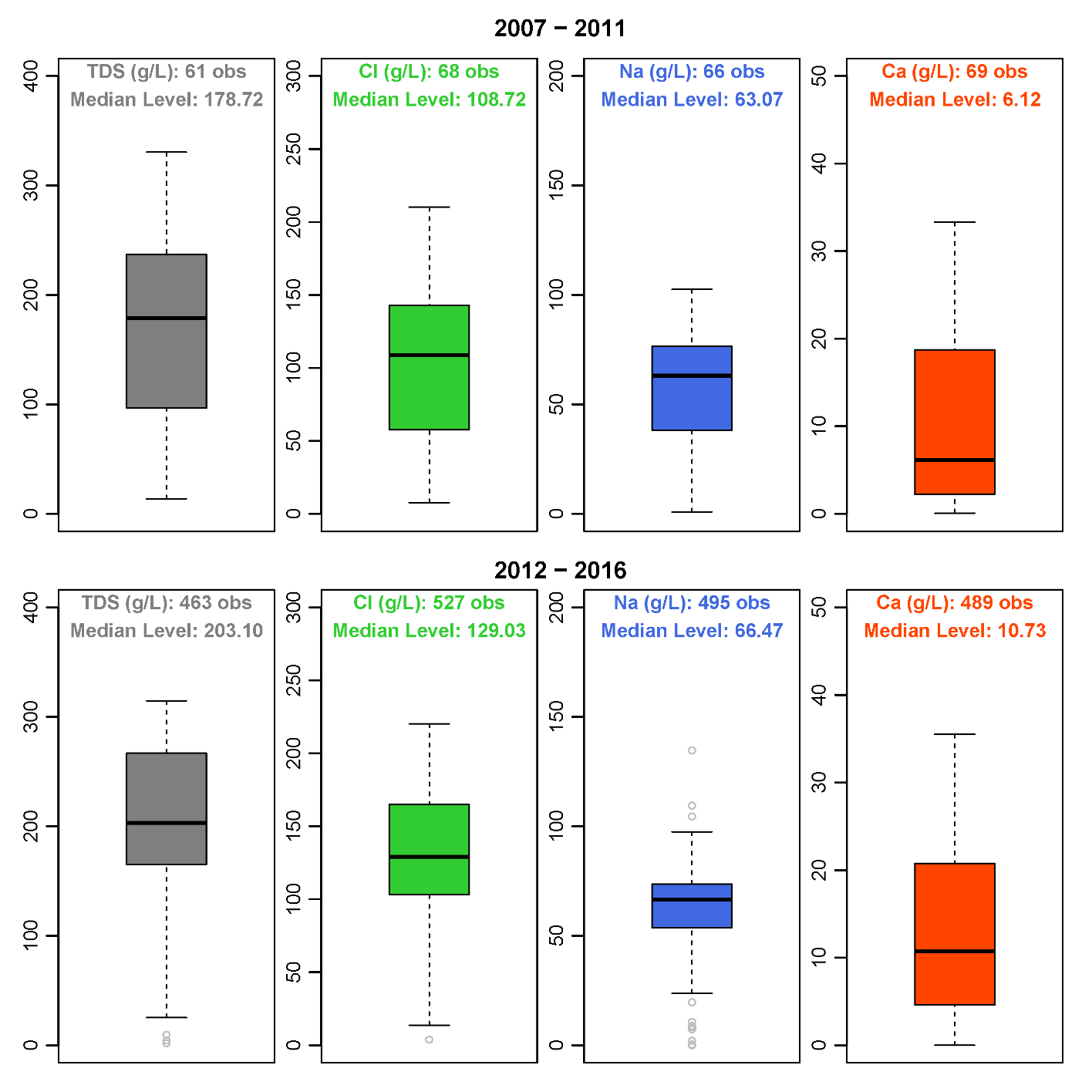


Supplementary Figure S1. The levels of TDS, Chloride, Sodium, and Calcium between different stages of shale development (2007-2011 v.s. 2012-2016). Note: The author produced the figure in R. The t-tests for the difference in means suggest that the increase in the level of concentration is statistically significant for all four constituents. The null hypothesis of no difference is rejected at the 95% confidence level in all cases (p values are 0.0012, 0.0001, 0.0513, 0.0062, respectively).
