# Supplementary material for: Shale oil production and groundwater: What can we learn from produced water data?
Source: PLoS One. 2021 Apr 30;16(4):e0250791. doi: 10.1371/journal.pone.0250791 (PMC8087075; doi:10.1371/journal.pone.0250791)
Supplement: S1 Table — (DOCX) [file pone.0250791.s002.docx]

**Supplementary Table S1. Summary statistics of variables (sample period: 2007-2016)**

| Variables | Sample mean | Standard deviation |
| --- | --- | --- |
| TDS concentration (mg/L) | 203,048 | 66,179 |
| *- Average (oil) well age* | 8.76 | 5.31 |
| *- Number of nearby oil wells (2-mile radius)* | 11.31 | 23.18 |
| *- Total annual oil production (Kbbl, 2-mile radius)* | 63.37 | 132.40 |
| *- Sample size* | 524 | |
| Chloride concentration (mg/L) | 127,363 | 41,296 |
| *- Average (oil) well age* | 8.63 | 5.08 |
| *- Number of nearby oil wells (2-mile radius)* | 10.93 | 22.52 |
| *- Total annual oil production (Kbbl, 2-mile radius)* | 60.47 | 125.05 |
| *- Sample size* | 595 | |
| Calcium concentration (mg/L) | 12,350 | 9,187 |
| *- Average (oil) well age* | 8.41 | 5.07 |
| *- Number of nearby oil wells (2-mile radius)* | 10.65 | 20.34 |
| *- Total annual oil production (Kbbl, 2-mile radius)* | 66.44 | 136.37 |
| *- Sample size* | 558 | |
| Sodium concentration (mg/L) | 62,055 | 17,836 |
| *- Average (oil) well age* | 8.47 | 4.72 |
| *- Number of nearby oil wells (2-mile radius)* | 11.13 | 21.70 |
| *- Total annual oil production (Kbbl, 2-mile radius)* | 65.79 | 133.64 |
| *- Sample size* | 561 | |
