# Supplementary material for: Shale oil production and groundwater: What can we learn from produced water data?
Source: PLoS One. 2021 Apr 30;16(4):e0250791. doi: 10.1371/journal.pone.0250791 (PMC8087075; doi:10.1371/journal.pone.0250791)
Supplement: S2 Table — (DOCX) [file pone.0250791.s003.docx]

**Supplementary Table S2: Regression of groundwater constituent levels on nearby oil production controlling well depth**

| **Constituent**  **Tested** | **Model Specification** | Total number of oil wells | | Total annual oil production | | |
| --- | --- | --- | --- | --- | --- | --- |
|  |  | (1) | (2) | | (3) | (4) |
|  | **Fixed Effects Dummy** | None | Geology + Year | | None | Geology + Year |
| TDS  (mg/L) | Number of oil wells | 310.7570***  (115.7277) | 305.1162***  (109.8299) | |  |  |
|  | Oil production (Kbbl) |  |  | | 30.0623  (20.4525) | 22.4342  (16.3865) |
|  | Average (oil) well age | 1412.4890***  (504.9047) | 1335.9830***  (483.5023) | | 1489.1470***  (509.7541) | 1388.1520***  (441.1219) |
|  | Well depth (foot) | 60.0646***  (7.1307) | 50.3210***  (6.9542) | | 60.6641***  (7.1635) | 51.1060***  (8.6315) |
|  | Sample size | 524 | | | | |
|  | Mean TDS (mg/L) | 203,048 | | | | |
|  | *R^2^* | 0.1461 | 0.2587 | | 0.1379 | 0.2495 |
| Chloride  (mg/L) | Number of oil wells | 232.2970***  (71.6576) | 221.5107***  (68.8132) | |  |  |
|  | Oil production (Kbbl) |  |  | | 8.3086  (13.0128) | 2.0019  (10.6649) |
|  | Average (oil) well age | 484.2621  (316.7179) | 301.2685  (307.7058) | | 532.3191*  (320.2233) | 321.8641  (393.0569) |
|  | Well depth (foot) | 32.7183***  (4.5901) | 26.5454***  (4.5485) | | 33.8846***  (4.6191) | 27.6456***  (4.8290) |
|  | Sample size | 595 | | | | |
|  | Mean chloride (mg/L) | 127,363 | | | | |
|  | *R^2^* | 0.1062 | 0.2032 | | 0.0909 | 0.1379 |
| Calcium  (mg/L) | Number of oil wells | 28.8733  (18.0080) | 22.6251  (17.4735) | |  |  |
|  | Oil production (Kbbl) |  |  | | -0.6540  (2.6979) | -1.0765  (2.2587) |
|  | Average (oil) well age | 240.5035***  (73.6481) | 355.7583***  (75.4782) | | 242.3447***  (74.0358) | 356.2941***  (85.5645) |
|  | Well depth (foot) | 6.1549***  (0.8717) | 7.3836***  (0.9023) | | 6.2116***  (0.8730) | 7.4150***  (1.1782) |
|  | Sample size | 558 | | | | |
|  | Mean calcium (mg/L) | 12,350 | | | | |
|  | *R^2^* | 0.1216 | 0.2020 | | 0.1176 | 0.1998 |
| Sodium  (mg/L) | Number of oil wells | 85.2746***  (34.5340) | 69.0405**  (33.0775) | |  |  |
|  | Oil production (Kbbl) |  |  | | 12.2581**  (5.6211) | 7.6181  (5.4305) |
|  | Average (oil) well age | 18.0572  (158.9481) | 85.3753  (155.1673) | | 59.0309  (159.4840) | 115.2649  (133.3002) |
|  | Well depth (foot) | 4.6031**  (2.0924) | 1.5253  (2.0374) | | 4.9509**  (2.0925) | 1.8083  (1.8189) |
|  | Sample size | 561 | | | | |
|  | Mean sodium (mg/L) | 62,055 | | | | |
|  | *R^2^* | 0.0205 | 0.1368 | | 0.0182 | 0.1331 |

Note: (1) Oil producing wells within a 2-mile radius (3.22 km) of the tested well are included in the analysis. (2) The heteroscedasticity-consistent standard error of each regression estimate is reported in the parentheses. Throughout the paper, asterisks (*, **, ***) indicate statistical significance at 10%, 5%, and 1% level, respectively, unless otherwise noted.
