# Supplementary material for: Shale oil production and groundwater: What can we learn from produced water data?
Source: PLoS One. 2021 Apr 30;16(4):e0250791. doi: 10.1371/journal.pone.0250791 (PMC8087075; doi:10.1371/journal.pone.0250791)
Supplement: Attachment — Submitted filename: PONE-D-21-02697_response_to_reviewers.pdf [file pone.0250791.s005.pdf]

Response to Reviewers

Manuscript ID: PONE-D-21-02697

Manuscript Title: Shale oil production and groundwater: What can we learn from produced water data?

Comments to the Author

Reviewer #1:

The author used the large data sample from the most productive portion of the Permian Basin – the four-county region in Southeast New Mexico between 2007 and 2016, to analyze the conditional correlations between shale oil production and produced water (PW) constituents. At the same time, he suggested that both produced water and its disposal should be regularly monitored and managed while incentivizing its reuse. Some specific comments are addressed in the following section.

Specific Comments:

1. The innovation of this article is not obvious enough. Please add three highlights before the abstract.

Response: Thank you for the suggestion. However, PLOS ONE style does not allow highlights. For your reference, here are three highlights of the paper:

- Data limitation prevents researchers from directly studying the deterministic geophysical and geochemical relationship between oil production and groundwater constituent levels. This study proposes an easy-to-implement alternative relying on existing produced water data and a fixed-effects linear regression model to shed some light on the relationship.
- This study shows that nearby drilling and oil production are found positively correlated with the levels of TDS, chloride, and sodium in PW samples. The intensity of drilling (measured by the number of wells) is the most relevant factor.
- The implication of their positive correlations with shale production can be significant because of the existing natural hydraulic connections between shallow aquifers and deep formations. The former are important sources for drinking water and irrigation water in the study region.

In addition, to highlight the contributions of this paper, the discussion and conclusion sections have been merged and re-written following other suggestions below. The last paragraph of the new section (Discussion and conclusions) highlights the contribution of the paper (please see the revised version, marked in red).

2. The process of processing oil production and groundwater constituent levels data, is not described clearly in Section 2.

Response: Thank you for the comment. The procedures of processing oil production data and groundwater data, including other relevant data information, have been further clarified in sub-section Study area and data (please see the revised version, marked in red).

3. The selection of the regression model lacks theoretic support. For example, in Eq.(1), it is assumed that there is a linear relationship between parameters , Controls, and . However, in practice, this may not be appropriate. Please clarify it.

Response: Thank you for your comments. The proposed linear regression model is used to explore the conditional correlation between oil production and groundwater constituent levels, not necessarily implying a deterministic causal relationship that indeed requires a geophysical and/or geochemical theory. It has been further clarified in sub-section Conditional correlation, paragraph 1 (please see the revised version, marked in red).

4. In this study, all the data were used for training without dividing the test set and the cross-validation set, and whether the model had overfitting problems was not discussed. Therefore, I am negative about the applicability and reliability of the regression model.

Response: Thank you for your comments. The regression model adopted in this study is used for the purpose of exploring conditional correlations rather than for prediction. Therefore, the out-of-sample predictability is not an evaluation criterion for the model. There is no need to conduct cross-validation by splitting the data sample into a training set and a test set. The statistical goodness of fit measure R^2 is enough for comparing different model specifications. It has been clarified in Results section, paragraph 1.

5. In Table 1, we see that the levels of TDS, Chloride, Calcium and Sodium correlate with the number of oil wells, oil production and average (oil) well age. Which of these three factors is dominant? I suggest that the author conduct a sensitivity analysis of these factors.

Response: Thank you. It is a good suggestion. The revised manuscript evaluates the contribution of each of these variables by conducting a variance decomposition based on the model goodness of fit R^2 (Huettner and Sunder, 2012). Specifically, we evaluate the change of R^2 by adding each of the concerned variables. The contribution of each independent variable is reported in percentage. The variable that contributes the most to R^2 is the dominant factor. Overall, the number of wells variable is dominant to the other two. The details are reported in the first paragraph of Results section (marked in red).

Reference:

Huettner, F., & Sunder, M. (2012). Axiomatic arguments for decomposing goodness of fit according to Shapley and Owen values. Electronic Journal of Statistics, 6, 1239-1250.

6. The discussion section is disappointing. The author only told us that since the onset of the shale development in the mid-2000s, nearby drilling and oil production were found positively correlated with the levels of TDS, chloride, and sodium in PW samples. However, readers are interested in why there is such a correlation. In addition, the author also pointed out many shortcomings of the regression model, which makes the results of this study seem immature.

Response: Thank you for the comment. As suggested by Reviewer #2, the discussion section and the conclusion section have been merged now. I agree that the geohydrological mechanisms behind the correlation are interesting to know. However, it is necessary to clarify that this study is positioned as an exploratory study to inspire further research. Currently, it is very challenging and cost-prohibitive to drill test wells at different depths and in a large enough spatial range to study it. We highlight such a research challenge in the new discussion and conclusion section (see the last paragraph, Section 4, marked in red).

The choice of methodology is constrained by the data availability. However, I believe it is critical to discuss the shortcomings of the chosen analysis method so that readers can interpret the results properly.

7. Conclusions drawn in this study is apparent.

Response: Thank you for the comment. Following the suggestion from Reviewer #2, the discussion section and the conclusion section have been merged. The revised manuscript has further elaborated on the implications of findings from this study. Please see the revised discussion and conclusions section.

Reviewer #2:

General Comment：

This manuscript explored the relationship between shale oil production and groundwater constituent levels in the Permian Basin. Produced water (PW) samples from active unconventional oil wells provided good reliability for the correlation relationship. The impact of shale gas/oil production on groundwater is an interesting topic. This manuscript also has a good review on the impact of shale oil/gas production on groundwater, but this manuscript did not discuss the long-term geochemical reaction of re-injected water in the reservoir. Valid conclusions are lacking and deserve to be discussed in depth. Thus, the reviewer can not recommend it for publication in the current form. A minor revision may be necessary. Following comments may be helpful:

Response: Thank you. Please see my point-by-point responses below on how to incorporate your suggestions.

Comment-1: The author pointed out the dramatic increase of the TDS, chloride, sodium, and calcium in the groundwater due to boom of shale gas/oil production. However, the mechanism of interaction between these elements is still unclear. Furthermore, what potential environmental hazards will be caused? Please explain it.

Response: Thank you for the comment. I agree that the geohydrological mechanisms behind the correlation are interesting to know. However, it is necessary to clarify that this study is positioned as an exploratory study to inspire further research. Currently, it is very challenging and cost-prohibitive to drill test wells at different depths and in a large enough spatial range to study it. We highlight such a research challenge in the new discussion and conclusions section. The most important potential environmental hazard is the contamination of freshwater aquifers, which affects sources of drinking water and irrigation water. The first paragraph of the new discussion and conclusions section has been revised to clarify these potential impacts (revision marked in red).

Comment-2: If the increase of the TDS, chloride, sodium, and calcium is an obvious trend, these related compounds play what’s role in geological formation? Please explain it.

Response: Thank you for the comment. I will try to explain it to the best of my knowledge. Salts that dissolved in the formation water are not valuable in terms of beneficial uses. However, they do help to maintain ion balance. A higher salinity level means that the solubility of CO2 decreases. Also, having really salty water can suppress microbes since it's hard to maintain a high osmotic gradient. It suggests that these salts inhibit hydrate formation. Meanwhile, if they can

help to suppress bacteria growth, then it prevents bacteria/microbes from decaying hydrocarbon reserves, at least in the periphery. It is worth noting that the role of these salts is not the focus of this study. We concern more about the potential impact of the upward movement of the saltier water on shallower groundwater aquifers.

Comment-3: The direction of Fig. 4 is not conducive to reading, it is recommended to retype.

Response: Thank you for the suggestion. The texts in the box plots are placed horizontally now in both Fig 4 and Fig S1.

Comment-4: The appendix files (Fig.S1 and Table S1) can not be found in the manuscript. Please check it.

Response: Thank you for pointing this out. The supplementary appendix file is attached separately. It requires separate downloading using the link on the last page of the manuscript file generated by the submission system.

Comment-5: The core of this manuscript is a linear regression formula (Eq.(1)), which lacks theoretical innovation. Please further explain the contribution of this manuscript.

Response: Thank you for your comments. The proposed linear regression model in equation (1) is used to explore the conditional correlation between oil production and produced water constituent levels, not necessarily implying a deterministic causal relationship. It has been further clarified in sub-section Conditional correlation (marked in red). The main contribution of the manuscript is to use existing produced water data to infer the potential impact of shale production on groundwater aquifers. The impact is difficult to measure directly. The contribution has been summarized in the concluding paragraph (Discussion and conclusions section, last paragraph).

Comment-6: It is recommended to merge the conclusion and discussion together.

Response: Thank you. The two sections have been merged as suggested.
