# Supplementary material for: Shale oil production and groundwater: What can we learn from produced water data?
Source: PLoS One. 2021 Apr 30;16(4):e0250791. doi: 10.1371/journal.pone.0250791 (PMC8087075; doi:10.1371/journal.pone.0250791)
Supplement: Attachment — Submitted filename: Review comment.doc [file pone.0250791.s004.doc]

**PLOS ONE**

Manuscript Number: PONE-D-21-02697

Title: Shale oil production and groundwater: What can we learn from large produced water datasets?

**Referee's comments**

**General Comment：**

This manuscript explored the relationship between shale oil production and groundwater constituent levels in the Permian Basin. Produced water (PW) samples from active unconventional oil wells provided good reliability for the correlation relationship. The impact of shale gas/oil production on groundwater is an interesting topic. This manuscript also has a good review on the impact of shale oil/gas production on groundwater, but this manuscript did not discuss the long-term geochemical reaction of re-injected water in the reservoir. Valid conclusions are lacking and deserve to be discussed in depth. Thus, the reviewer can not recommend it for publication in the current form. A minor revision may be necessary. Following comments may be helpful:

**Comment-1:** The author pointed out the dramatic increase of the TDS, chloride, sodium, and calcium in the groundwater due to boom of shale gas/oil production. However, the mechanism of interaction between these elements is still unclear. Furthermore, what potential environmental hazards will be caused? Please explain it.

**Comment-2:** If the increase of the TDS, chloride, sodium, and calcium is an obvious trend, these related compounds play what’s role in geological formation? Please explain it.

**Comment-3:** The direction of Fig. 4 is not conducive to reading, it is recommended to retype.

**Comment-4:** The appendix files (Fig.S1 and Table S1) can not be found in the manuscript. Please check it.

**Comment-5:** The core of this manuscript is a linear regression formula (Eq.(1)), which lacks theoretical innovation. Please further explain the contribution of this manuscript.

**Comment-6:** It is recommended to merge the conclusion and discussion together.
